# Repeatability and reliability of retinal arterial hemodynamics measurement using Doppler holography

**DOI:** 10.1117/1.JBO.31.4.046001

**Published:** 2026-03-24

**Authors:** Olivier R. Martinache, Robert L. Draham, Valerie C. Snyder, Jay Chhablani, José-Alain Sahel, Ethan A. Rossi, Michael Atlan

**Affiliations:** a Paris Sciences & Lettres (PSL) University, Langevin Institute – CNRS. École Supérieure de Physique et de Chimie Industrielles (ESPCI) Paris, Paris, France; b University of Pittsburgh, Department of Ophthalmology, Pittsburgh, Pennsylvania, United States; c UPMC Vision Institute, Pittsburgh, Pennsylvania, United States; d University of Pittsburgh, Department of Bioengineering, Swanson School of Engineering, Pittsburgh, Pennsylvania, United States

**Keywords:** retinal imaging, blood flow, Doppler holography, lasers, repeatability, reliability

## Abstract

**Significance:**

Reliable quantification of retinal arterial blood flow is important for diagnosing and monitoring ocular and systemic diseases. Existing techniques are limited by invasiveness, motion artifacts, or a lack of quantitative flow estimation.

**Aim:**

The aim is to assess the repeatability, reproducibility, and robustness of laser Doppler holography (LDH) for measuring retinal arterial hemodynamics.

**Approach:**

We acquired LDH data at 67 kHz in healthy volunteers (14 eyes intra-day and 4 eyes inter-day) and quantified blood volume rate, resistivity index (RI), and vessel diameter. Additional measurements evaluated sensitivity to axial displacement and gaze lateral positioning.

**Results:**

LDH successfully measured retinal arterial blood volume rate in all eyes, with a coefficient of variation (CoV) of 18.5% for the mean arterial blood volume rate and a CoV of 11% for RI. Inter-day reproducibility remained acceptable (CoV≈20%). The mean arterial diameter estimation showed a CoV of <3%. Moderate axial or lateral shifts introduced small changes in hemodynamic values (<15% CoV) compared with inter- or intra-day tests.

**Conclusions:**

LDH provides reliable and robust measurements of retinal arterial hemodynamics and maintains performance under typical imaging variations (axial or gaze position). These findings support its potential for longitudinal studies and future clinical translation.

## 1 Introduction

The precise determination of the retinal blood volume rate (BVR) carries great clinical promise, as it can provide valuable information for the diagnosis, observation, and treatment of various retinal and systemic diseases. Identifying patients at certain risk for sight-threatening complications relies heavily on the early detection of changes in retinal blood flow. In the cases of diabetic retinopathy^1^^–^^3^ and glaucoma,^4^^,^^5^ these abnormalities can indicate disease progression, underscoring the importance of BVR monitoring for prompt intervention and treatment strategy development. Similarly, age-related macular degeneration, which is a leading cause of vision loss, shows hemodynamic changes.^6^^,^^7^ Elucidating these changes may shed light on the pathophysiology of the disease. Beyond ocular health, the retina’s vascular dynamics reflect broader systemic conditions, such as hypertension,^8^^,^^9^ where retinal BVR measurements can serve as an indicator of cardiovascular health.^10^

A variety of imaging techniques have been developed in clinical ophthalmology to meet the increasing demand for enhanced visualization, quantification, and functional assessment of retinal perfusion.^11^ Fluorescein angiography,^12^ the gold standard in this field, enables dynamic visualization of vascular leakage,^13^ neovascularization,^14^ and non-perfusion.^15^ However, it necessitates dye injection and has limited depth resolution. Using near-infrared wavelengths, indocyanine green angiography broadened this imaging technique to encompass the deeper choroidal circulation, facilitating improved diagnosis of conditions such as polypoidal choroidal vasculopathy.^16^ Both methods are, nonetheless, qualitative and invasive. Non-Doppler, high-resolution methods allow the retinal flow imaging approach to be applied at the microvascular scale. Adaptive optics (AO) line-scan systems^17^^,^^18^ make use of the streaks left by red blood cell (RBC) motion to calculate velocity in small vessels. Yet, clinical translation remains limited by system complexity and field of view. Optical coherence tomography (OCT)-angiography^19^ uses RBC motion contrast to create maps of vessels where flow is pumping, making it suitable for microvascular network perfusion mapping without providing quantitative flow data.

Doppler-based methods represent a significant advance in functional flow imaging in large vessels. With laser Doppler velocimetry, it became possible to make absolute, non-invasive measurements of blood velocity in individual large-caliber retinal vessels for the first time.^20^^,^^21^ Conversely, color Doppler ultrasound imaging (CDI) can assess flow in major ocular arteries such as the central retinal artery (CRA) and ophthalmic artery, providing insight into retrobulbar circulation.^22^^–^^25^ Nonetheless, the clinical use of CDI is limited due to the intensity limits of retinal regulation and insufficient spatial resolution. Complementary techniques such as laser speckle flowgraphy^26^ and laser Doppler flowmetry^27^^–^^29^ made it possible to map relative blood flow in two dimensions at localized points and across large retinal areas, respectively. More recently, Doppler optical coherence tomography (DOCT)^30^^–^^33^ has made significant strides in the field by combining structural imaging and phase-resolved Doppler assessment. However, issues such as motion artifacts and angular dependency prevent DOCT from being widely clinically used.

By integrating holography with the Doppler effect, laser Doppler holography (LDH) emerges as a promising technique for the quantitative assessment of retinal blood flow in the primary retinal arteries.^34^ LDH offers remarkable temporal resolution and adequate spatial resolution, enabling the capture of high-frame-rate retinal blood flow data, even amidst the pulsatile phases of the cardiac cycle (systolic peak and dicrotic notch).^35^

This study seeks to evaluate the reliability and repeatability of the LDH technique for the quantitative assessment of BVR in the retinal vasculature using an offline ultrafast camera. The investigation focuses on measurement consistency through intra-day and inter-day comparisons as well as axial position and gaze direction impact on biomarker estimation.

## 2 Materials and Methods

### 2.1 Participants

Informed consent was obtained from all subjects. The experiments were approved by the University of Pittsburgh Institutional Review Board and adhered to the tenets of the World Medical Association Declaration of Helsinki (Ethical Principles for Medical Research Involving Human Participants). Fifteen eyes (either right or left) from healthy volunteers were evaluated using the LDH, resulting in a dataset of 170 hemodynamic measurements, by session of 4 consecutive measurements (except for axial and lateral positioning studies) lasting ∼15  min in total. Measurements were done in a low-light room ambiance. No dilation drops were used in this study, and head stabilization was ensured with a custom-engineered chinrest.^36^ Eye fixation was stabilized by directing the fellow eye (contralateral to the eye under examination) toward a three-dimensional-printed fixation target that was optically conjugated to the same eyepiece employed in the sample arm, thereby maintaining consistent gaze alignment during measurement.

### 2.2 Laser Doppler Holography Setup

Doppler holography employs 852-nm laser light to perform interferometric imaging of the retina, integrating a Mach–Zender in-line holographic setup,^37^ which captures both the amplitude and phase of reflected light waves, with the Doppler effect to detect frequency shifts induced by motion of blood cells. A high-speed imaging sensor records the resulting interference pattern, generating high-contrast images of endoluminal blood flow. Two key characteristics of Doppler holography are its lack of depth sectioning, enabling choroidal visualization, and its diffuse illumination, allowing a wide field of view. The volunteer’s eye positioning was monitored through real-time visualization of inline digital holograms of the eye fundus, generated from interferograms recorded with an Adimec Quartz Q-2A750-Hm/CXP-6 camera (pixel size 12  μm, 1024×1024  pixels). The software Holovibes (release 13.2.3) was used for real-time rendering of holograms. Data for this study were collected using an ultrahigh-speed offline camera (Ametek - Phantom V2012, 12-bit pixel depth, pixel size 28  μm), with a 16-bit, 512×512  pixel interferogram stream recorded at 67 kHz. The offline reconstruction streamline is composed of angular spectrum transformation, singular value decomposition filtering, and short-time Fourier transformation applied within 512-frame windows with the HoloDoppler (release 1.2) digital hologram software. A total of 4 (2 left eyes and 2 right eyes) 187,000 raw interferogram frames, amounting to 4×78  GB of data, were acquired offline for image rendering and analysis with EyeFlow (release 2.6). All software and algorithms are available on the GitHub repository (https://github.com/DigitalHolography). The imaging protocol was focused on centering the optic nerve head (ONH) and its peripapillary regions, distinctly visualizing the principal branches around the CRA responsible for retinal perfusion, as well as the central retinal vein, responsible for retinal drainage.

### 2.3 Methods

**Fig. 1:**
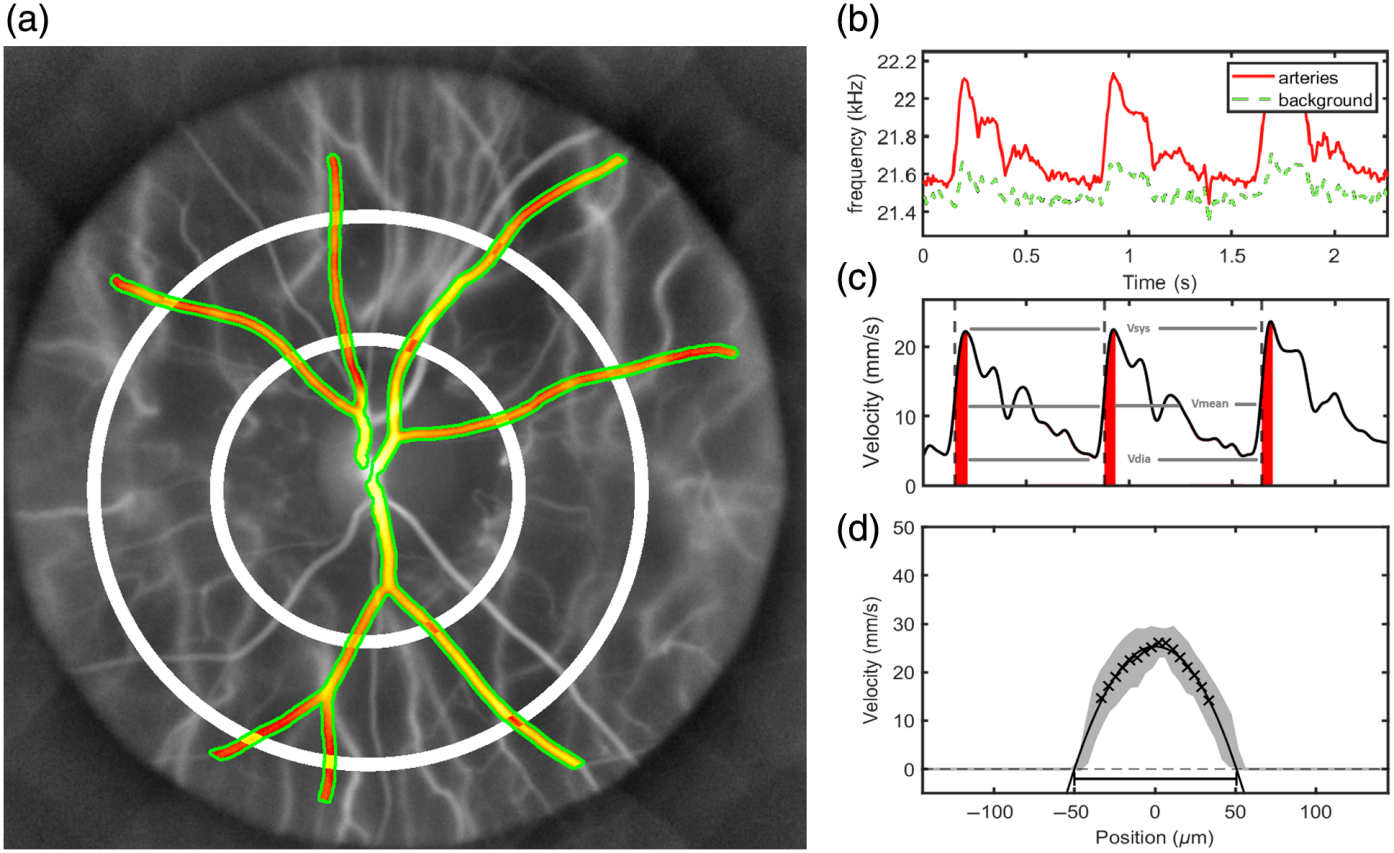
(a) Time-integrated hemodynamic velocity map showing segmented arterial regions (red), surrounding background tissue (green), and white annular regions used for spatial averaging. Spatially averaged Doppler frequency broadening measured in arterial regions (red) and background tissue (green) (b). Arterial waveform showing mean, systolic, and diastolic velocity, obtained by subtracting background from arterial frequency broadening, applying Eq. (1), and low-pass filtering over an integer number of cardiac cycles. The dark red area indicates the arterial stroke volume (c). Representative mean arterial velocity profile of a typical section used for diameter estimation (d).

Our method for retinal blood flow quantification follows a two-stage process:

1. Primary in-plane retinal arteries were segmented [Fig. 1(a)] through a series of algorithms: Frangi segmentation,^38^ temporal correlation, Otsu thresholding,^39^ and choroidal vessel removal based on anatomical features. Manual adjustment of these masks was performed when choroidal vessels were still detected to assess only the retinal vasculature to ensure blood flow analysis of identical vessels among consecutive measurements. The differential Doppler frequency broadening δf between the arterial vessels and surrounding tissue was measured by calculating the square root of the absolute difference among their normalized second-order Doppler spectrum moments. This analysis was conducted within the frequency range of 6 to 33 kHz. The resulting value was adjusted by multiplying by +1 or −1, depending on the sign of the differential, to correct for discrepancies where the Doppler broadening in the local neighborhood did not accurately reflect the arterial background signal.
2. A forward scattering physical model, based on a diffused secondary light source in deeper retinal layers, was employed to derive the local root mean square (RMS) blood flow velocity v in in-plane retinal arteries from the estimated local increase in Doppler broadening in retinal arteries. This local velocity was calculated as the product of the optical wavelength (λ=852  nm) and the local differential Doppler broadening (Δf), divided by the estimated eye numerical aperture NA = 0.25.

This procedure follows early work reported in Fischer et al.,^34^ demonstrating estimation of absolute blood flow quantification using a forward-scattering model at a frame rate of 33 kHz with a real-time camera (S711 phantom). Measurements acquired at a higher frame rate of 67 kHz, implemented to mitigate Doppler signal clipping related to the Nyquist cutoff frequency and obtained using an offline camera (v2012 phantom), revealed discrepancies in the estimated blood velocity, indicating that further refinement of the model is required. In particular, the velocity estimation was found to depend on acquisition parameters such as laser power, the spectral bandwidth used for time-frequency analysis, eye–camera distance, and camera characteristics including exposure time, quantum efficiency, pixel size, and dynamic range. Although the real-time setup yields blood flow estimates consistent with values reported using other techniques when applying a forward-scattering model, the offline configuration systematically produces lower velocity estimates. To account for this difference, a calibration factor α=5 was applied to align the mean total arterial blood volume rate to 39  μL/min across the 15 subjects included in this study. This scaling factor modifies the proportionality coefficient relating Δf to v but does not affect the linearity^40^ of Eq. (1) nor the primary objective of this work, which is to assess the reliability and repeatability of LDH-derived hemodynamic measurements

$$v=\frac{\alpha \lambda \Delta f}{\mathrm{NA}}.$$ (1)

From this equation, velocity profiles can be mapped to compute vessel diameter via Poiseuille law fitting, as illustrated in [Fig. 1(b)]. This cardiac profile is plotted by averaging the velocity profile in principal retinal arteries of about the same caliber (80 to 120  μm typically).

Local estimation of absolute blood volume rates is computed by multiplying the local velocity [Eq. (1)] and by the cross-sectional area at multiple sections along each segmented arterial branch. Averaging across sections improves the signal-to-noise ratio of the flow estimation within each branch, and the total arterial blood volume rate is obtained by summing the contributions of all branches.

Regarding the measurement of arterial diameter for section estimation, it is known to vary over the cardiac cycle, which affects blood volume rate estimation. However, previous studies such as in Senée et al.^41^ have reported limited diameter pulsatility in large retinal arteries, on the order of ±2% over the cardiac cycle. Given the small magnitude of these variations relative to measurement noise and inter-subject variability, vessel diameter was estimated by averaging the velocity profile over the entire cardiac cycle. Although vessel diameter enters quadratically in the calculation of blood volume rate, a ±2% diameter variation would result in a flow variation below ±4%, which remains smaller than the repeatability limits reported in this study. Therefore, dynamic diameter changes were neglected in the present analysis to favor robustness and signal-to-noise ratio. To obtain the pixel size in the image plane, the optic disc was taken as a reference, assuming its horizontal diameter to be 1.79±0.27  mm.^42^

The mean, systolic, and diastolic blood volume rates (μL/min) were determined from the arterial volume rate waveform over the cardiac cycle. Arterial stroke volume (nL) was calculated by integrating the area under the BVR curve between the maximum derivative and the systolic peak as displayed in Fig. 1(c). The resistance index (RI, Pourcelot index) and the pulsatility index (Gosling index), which quantify the modulation depth of the cardiac cycle,^43^ were estimated from both velocity and blood volume rate waveforms according to Eq. (2), thereby characterizing the variation of velocity/blood volume rate over the cardiac cycle relative to the mean or peak values. Heartbeat and mean diameter were also evaluated in beats per minute (BPM) and micrometers (μm), respectively

$$\mathrm{RI}=\frac{v_{\text{systolic}}-v_{\text{diastolic}}}{v_{\text{systolic}}},\;\mathrm{PI}=\frac{v_{\text{systolic}}-v_{\text{diastolic}}}{v_{\text{mean}}}.$$ (2)

### 2.4 Protocol

**Fig. 2:**
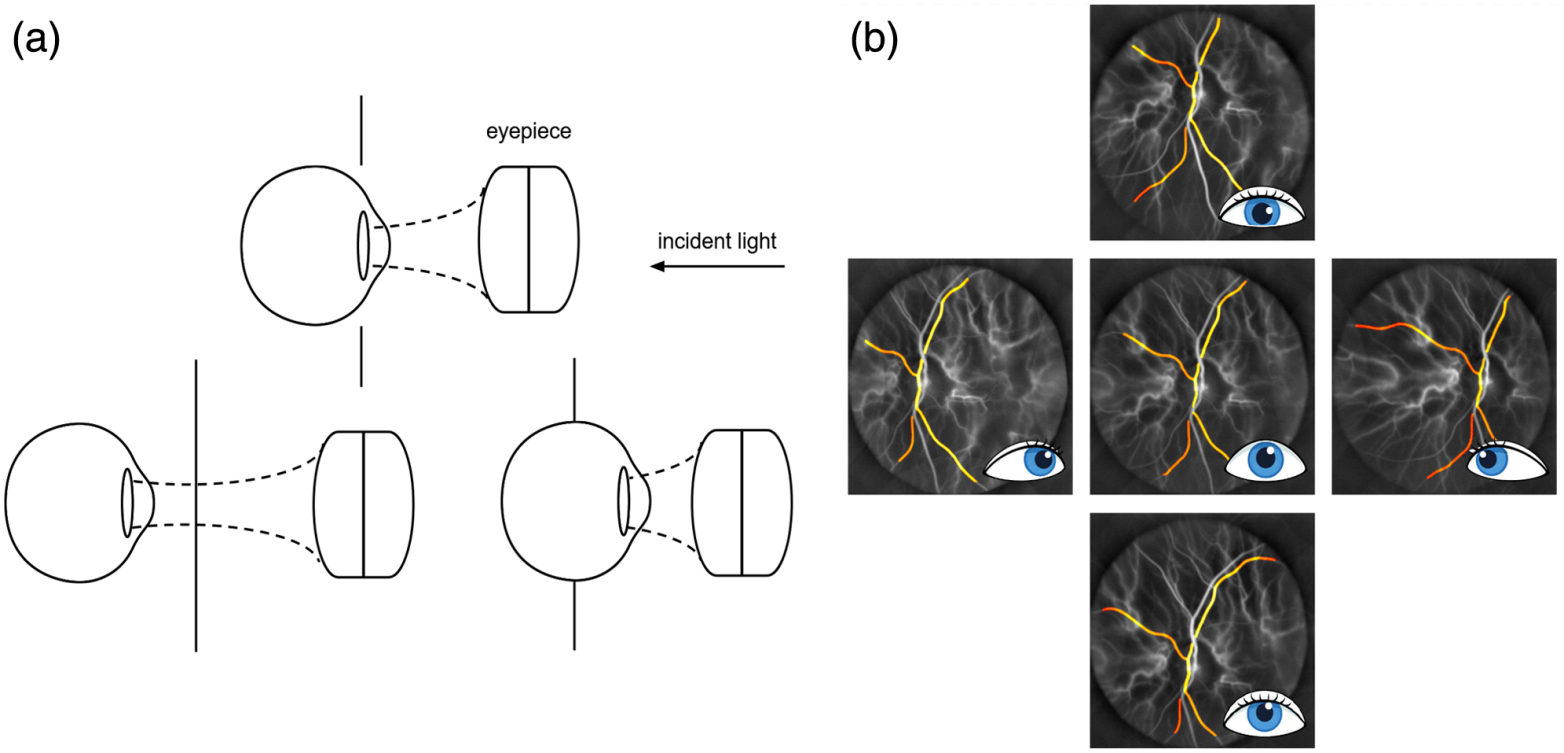
(a) Effect of axial eye alignment on post-eyepiece optical throughput, sampled at ten positions around the optimal (marked with a vertical line) as explained in Sec. 2.4, the optimal position being the reference alignment for all measurements. (b) Power Doppler images acquired at five ONH lateral positions corresponding to different gaze directions to assess LDH robustness, with the same retinal vessels segmented.

A nominal optical output power of up to 4 mW at a wavelength of 852 nm was employed in a diffuse Maxwellian view configuration.^44^ Irradiance at the cornea and the crystalline lens was $20\;\mathrm{mW}/{\mathrm{cm}}^{2}$. Irradiance at the posterior segment (retina) was $29\;\mathrm{mW}/{\mathrm{cm}}^{2}$. This irradiation level is compliant with the exposure level of the international standard for ophthalmic instruments ISO 15004-2:2024 and the American National Standards Institute ANSI Z80.36-2021. This study was conducted to evaluate the repeatability and robustness of BVR across these different cases:

• Intra-day study was conducted on 14 eyes of 7 subjects, imaged at 3 points throughout the day (morning 9 to 10 am, 1 h pre- or post-prandial—around 12 pm and afternoon 3 to 4 pm). Each eye was imaged twice at each time point to evaluate the repeatability of measurements along 1 day, amounting to 6.5 TB of data.
• Inter-day investigation was performed on four eyes from two subjects, imaging sessions were conducted in the morning timeframe (9 to 10 am), across eight separate days within a 3-week interval. Each eye underwent two acquisitions per session to assess inter-day measurement repeatability, amounting to 10 TB of data.
• Axial positioning test was performed on 1 eye, acquiring 10 measurements spaced 2 mm apart using a micrometer stage. The first and last positions corresponded to the closest and farthest from the eyepiece, with the 5th considered optimal for maximal pupil illumination [Fig. 2(a)]. This test assessed the effect of axial displacement, influenced by operator alignment and forehead-to-eye distance, generating 1.3 TB of data.
• Fixation positioning of the optic nerve head study was conducted on one eye, an imaging session consisting in five measurements each with a different gaze direction [Fig. 2(b)]. This amounted to 0.5 TB of data.

All these 163 measurements, done on 15 different healthy eyes, sum up to around 18 TB of data. Only datasets measuring several cardiac cycles were included in the repeatability analysis; 10 out of the 163 measurements were removed from the study related to blinking during acquisition, thereby avoiding systematic bias arising from comparisons between non-averaged and averaged measurements.

## 3 Results

### 3.1 Intra- and Inter-Day Study

**Fig. 3:**
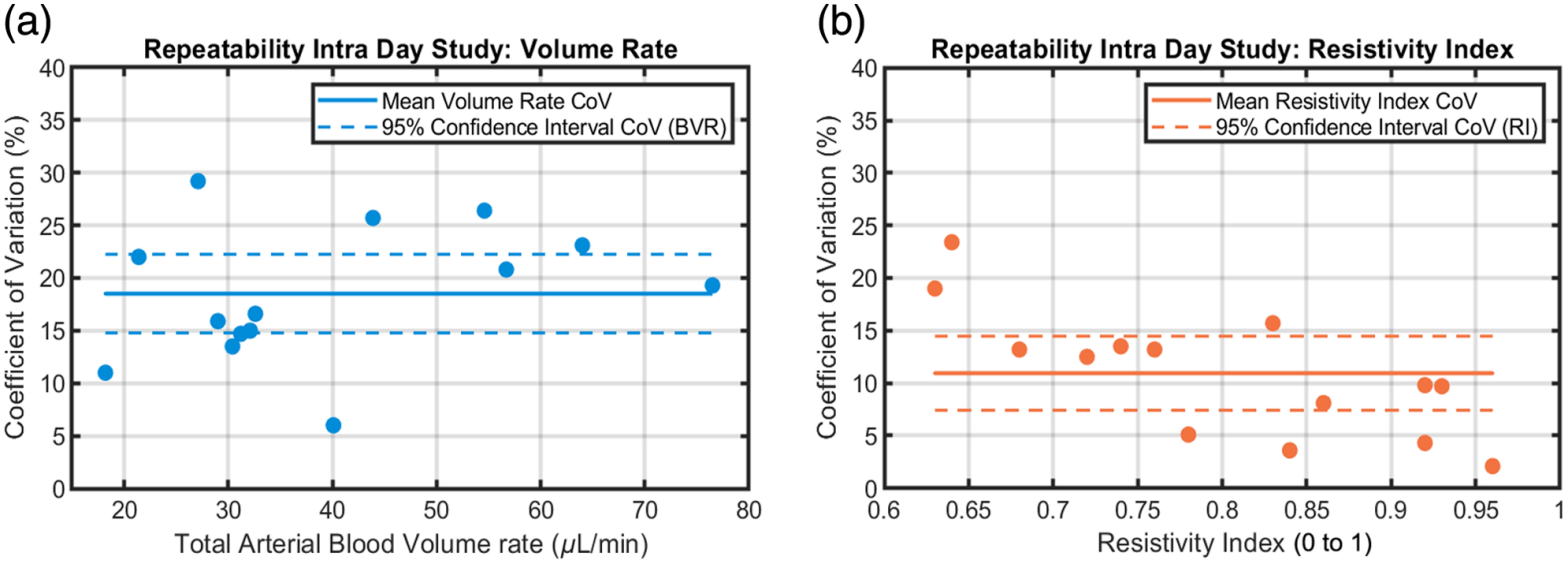
(a) Total arterial volumetric flow rates plotted against their CoV. Each point is the mean of six measurements acquired across three time intervals (morning, midday, and afternoon), excluding blink-corrupted data. The solid line shows the mean CoV (18.5%), and the dashed lines indicate the 95% CI (14.8 to 22.2%). (b) CoV values of the resistivity index, with the solid line marking the mean CoV (10.9%) and the dashed lines showing the 95% CI (7.3 to 14.5%).

Intra-day repeatability values for the mentioned hemodynamic biomarkers are presented in Table 1. The mean and systolic total retinal volume rate data $Q_{M}$ and $Q_{S}$ presented good repeatability results with a mean coefficient of variation (CoV) equal to 18.5% and 18.1%, respectively. The total arterial and volumetric flow rates exhibit substantial intersubject variability, ranging from ∼18 to 44  μL/min. This could be explained by the considerable differences in total arterial cross-section as explained by Riva et al.^20^ Arterial resistivity, whether derived from velocity or volumetric flow rate profiles, demonstrates a high degree of consistency: mean ${\mathrm{RI}}_{V}=0.80$ with CoV = 10.9% and mean ${\mathrm{RI}}_{Q}=0.76$ with CoV = 12.9%. Pulsatility index also displays comparable results between ${\mathrm{PI}}_{V}$ and ${\mathrm{PI}}_{Q}$ but with higher CoV of around 25%. The mean diameter demonstrates the highest intra-session reliability with a mean of ρ=83.7  μm and a CoV of 2.7%. Conversely, $Q_{D}$ and $V_{\mathrm{AS}}$ exhibit poor repeatability, due to low signal-to-noise ratio (SNR) during the diastole end cycle and the impact of the noise on the timestamp estimation of the arterial stroke volume. Total arterial volumetric flow rate and velocity-based resistivity index were plotted as a function of CoV in Fig. 3. The 95% confidence interval (CI) is calculated using the two-tailed t-distribution (n=14 eyes, 7 subjects), which gives a coefficient t0.975≈2.160, used in the equation CI95%=mean±t0.975×Standard Error of the Mean.

**Table 1:** Hemodynamic parameters estimation for the intra-day study (14 eyes), including systolic, diastolic, and mean total volume rates ($Q_{S}$, $Q_{D}$, and $Q_{M}$), arterial stroke volume ($V_{\mathrm{AS}}$), velocity- and volume-based resistivity and pulsatility indexes (${\mathrm{RI}}_{V}$, ${\mathrm{PI}}_{V}$, ${\mathrm{RI}}_{Q}$, and ${\mathrm{PI}}_{Q}$), heartbeat H, and mean arterial diameter ρ. Metrics are reported as mean, SD, and CoV over six measurements per subject, with ±1σ indicating group variability.

	Mean	SD	CoV
$Q_{S}$ (μL/min)	77.5 ± 42.0	12.7 ± 6.0	18.1 ± 6.2%
$Q_{D}$ (μL/min)	14.4 ± 5.2	6.8 ± 4.7	46.6 ± 28.4%
$Q_{M}$ (μL/min)	39.8 ± 16.5	7.7 ± 4.6	18.5 ± 6.2%
$V_{\mathrm{AS}}$ (nL)	169.4 ± 44.8	55.3 ± 25.7	33.5 ± 14.0%
${\mathrm{RI}}_{v}$	0.80 ± 0.11	0.08 ± 0.04	10.9 ± 5.9%
${\mathrm{PI}}_{v}$	1.64 ± 0.50	0.39 ± 0.19	24.9 ± 9.9%
${\mathrm{RI}}_{Q}$	0.76 ± 0.11	0.09 ± 0.04	12.9 ± 6.8%
${\mathrm{PI}}_{Q}$	1.49 ± 0.47	0.37 ± 0.15	26.0 ± 9.8%
H (bpm)	95.1 ± 8.8	15.5 ± 6.8	16.5 ± 7.3%
ρ (μm)	84 ± 13	3 ± 1	2.7 ± 1.0%

**Fig. 4:**
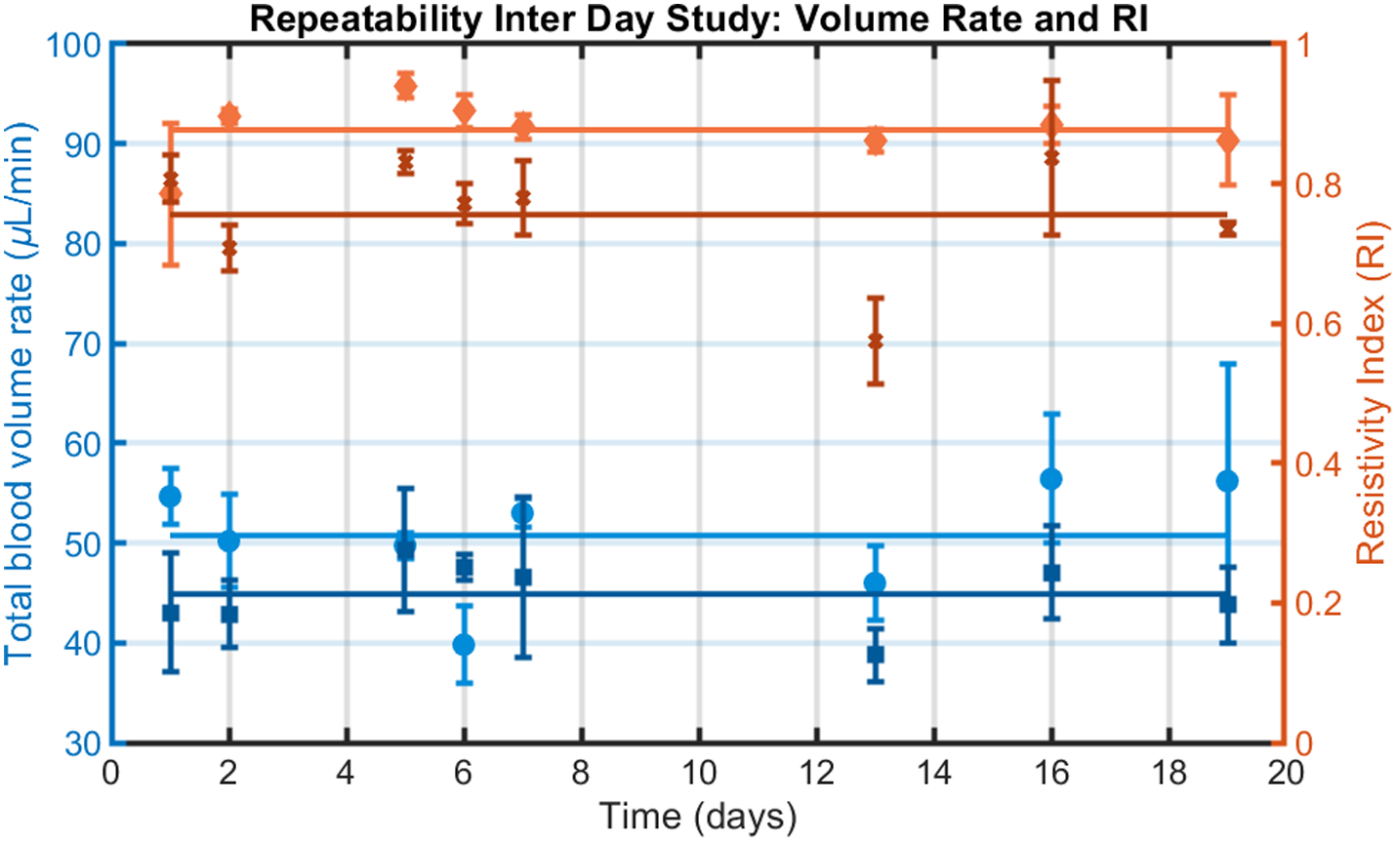
Total arterial volume rate (blue) and velocity-based resistivity index (orange) in both eyes of a normal subject measured over 20 days for the inter-day study. Each time point is the mean of two consecutive measurements, plotted as mean ± standard deviation (SD). The left and right eyes are respectively in light and dark colors.

Inter-day reproducibility results are presented in Fig. 4, illustrating the total blood volume rate and resistivity index values of the left and right eyes of one healthy subject over a 20-day interval. Confidence interval coefficient is t0.975≈2.365 (n=8). Similar outcomes were observed in the other subject. The averaged results over the two subjects are presented in Table 2.

**Table 2:** Estimation of hemodynamic parameters for the inter-day study (four eyes of two healthy subjects, over a 20-day interval), comprising the same metrics as explained in Table 1.

	Mean	SD	CoV
$Q_{S}$ (μL/min)	103.3 ± 15.8	23.1 ± 10.2	21.8 ± 7.4%
$Q_{D}$ (μL/min)	13.8 ± 4.9	7.2 ± 1.8	60.7 ± 27.1%
$Q_{M}$ (μL/min)	47.1 ± 3.1	9.7 ± 3.0	20.5 ± 6.1%
$V_{\mathrm{AS}}$ (nL)	216.3 ± 6.5	63.5 ± 17.4	29.6 ± 8.9%
${\mathrm{RI}}_{v}$	0.88 ± 0.07	0.08 ± 0.01	9.0 ± 2.5%
${\mathrm{PI}}_{v}$	2.06 ± 0.45	0.44 ± 0.12	21.4 ± 3.0%
${\mathrm{RI}}_{Q}$	0.85 ± 0.08	0.09 ± 0.02	10.3 ± 2.9%
${\mathrm{PI}}_{Q}$	1.92 ± 0.45	0.42 ± 0.12	22.1 ± 2.6%
H (bpm)	86.5 ± 4.5	17.5 ± 2.9	20.1 ± 2.4%
ρ (μm)	99 ± 10	3 ± 1	2.8 ± 0.4%

Inter-day measurements generally show higher variability than intra-day measurements (CoV higher in 8 out of 10 metrics), likely reflecting physiological fluctuations in blood flow due to factors such as stress, fatigue, thermoregulation, or visual stimulation.^45^^,^^46^ These findings should be confirmed with additional measurements across a larger cohort.

Nevertheless, reproducibility across sessions is acceptable, as indicated by a total BVR of 47.1±3.1  μL/min with a CoV of 20.5±6.1%. The same result for the resistivity index has an average of 0.88 and 0.85 for velocity and BVR-based calculation with CoV of 9.0 and 10.3%.

### 3.2 Axial and Lateral Study

**Fig. 5:**
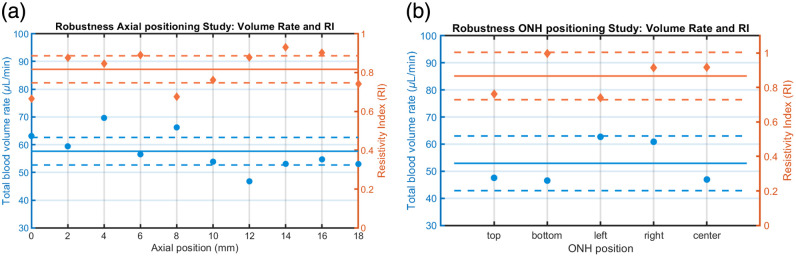
Blood volume and velocity-based RI measurements across different eye positions for the axial positioning and ONH lateral positioning studies, showing overall mean (solid line) and 95% CI (dotted lines) for both metrics. (a) Axial positioning study: total BVR (blue) and velocity-derived RI (orange) across an 18-mm axial range. Mean BVR: 57.6  μL/min (95% CI: 52.7 to 62.6) and RI: 0.82 (95% CI: 0.74 to 0.89). (b) ONH lateral positioning study: total BVR (blue) and velocity-based RI (orange) at five lateral positions. Mean BVR: 52.9  μL/min (95% CI: 42.8 to 63.0) and RI: 0.87 (95% CI: 0.73 to 1.00).

Axial positioning reliability was investigated by repeated LDH measurements at different axial positions across an 18-mm span (10 measurements spaced by 2 mm). 95% confidence interval coefficient was then equal to t0.975≈2.262 (n=10). Data acquisitions were unique for one given position, due to the offline setup necessitating data transfer which introduced time delays. Therefore, measurements were performed within a limited timeframe to minimize temporal variability. Results from one left eye are in Table 3. The total volume rate $Q_{M}$ exhibits small variation along the axial extent, yielding a mean value of 57.6±6.9  μL/min (12.0% CoV) and a resistivity index of 0.82±0.10 (12.2% variation), as displayed in Fig. 5. Among all studies, arterial stroke volume demonstrated the highest measurement reliability in this axial test. This is most likely attributed to data acquisition from a single eye with sufficient SNR to ensure accurate time estimation of cardiac cycle events, resulting in a reproducibility of 226.0±28.0  nL (12.4% variation). In summary, the axial shift produced results comparable to those obtained at the optimal focal plane, suggesting that axial misalignment exerts only a minor impact on the estimation of vascular metrics.

**Table 3:** Hemodynamic parameters for the axial and lateral positioning studies, based on 10 and 5 measurement positions of the same eye, respectively. The metrics match those reported in the previous tables, and values are expressed as mean ± SD (CoV).

	$Q_{S}$ (μL/min)	$Q_{D}$ (μL/min)	$Q_{M}$ (μL/min)	$V_{\mathrm{AS}}$ (nL)	${\mathrm{RI}}_{V}$	${\mathrm{PI}}_{V}$	${\mathrm{RI}}_{Q}$	${\mathrm{PI}}_{Q}$	H (bpm)	ρ (μm)
Axial	105.7 ± 17.0 (16.1%)	22.4 ± 7.8 (34.8%)	57.6 ± 6.9 (12.0%)	226.0 ± 28.0 (12.4%)	0.82 ± 0.10 (12.2%)	1.61 ± 0.40 (24.8%)	0.78 ± 0.09 (11.5%)	1.46 ± 0.36 (24.7%)	99 ± 27 (27.3%)	88 ± 2 (2.4%)
Lateral	115.4 ± 21.5 (18.6%)	16.3 ± 9.3 (57.1%)	52.9 ± 8.1 (15.3%)	229.0 ± 76.0 (33.2%)	0.87 ± 0.11 (12.6%)	2.09 ± 0.76 (36.4%)	0.85 ± 0.10 (11.8%)	1.91 ± 0.64 (33.5%)	109 ± 35 (32.1%)	124 ± 4 (3.0%)

The ONH positioning robustness study aims to determine the impact of the lateral setting between the LDH illumination and the gaze direction on LDH vascular metric estimation. The five measurements impose a 95% CI coefficient t0.975≈2.776. The results about the BVR and resistivity are displayed in Fig. 5 and extensive data developed in Table 3.

The total volume rate shows limited variation across the lateral ONH position, with a mean value of 52.9±8.1  μL/min (15.3% CoV) and a resistivity index of 0.87±0.1 (12.6% CoV). The mean vessel diameter maintained high reliability in this assessment, measured at 124±4  μm (3.0% CoV). In summary, the lateral positioning results did not significantly differ from repeatability measurements at the same location, indicating that lateral displacement exerts a negligible effect on the estimation of vascular metrics.

## 4 Discussion

By employing an offline imaging setup capable of capturing interferometric data at 67 kHz, we were able to achieve high temporal resolution retinal blood flow assessment, essential for resolving fast hemodynamic events such as the systolic peak and dicrotic notch within the cardiac cycle. These results lay the groundwork for real-time implementations of the technique, using high-speed cameras operating at 33 kHz, approaching the threshold necessary for accurate Doppler broadening estimation in principal retinal vessels. Achieving sufficient temporal sampling is crucial to prevent aliasing effects and signal clipping that may occur when the Doppler shift exceeds the Nyquist frequency during phases of high blood velocity, particularly during the systolic peak. The current offline setup avoids such artifacts.

Additional acquisition parameters such as laser intensity, camera exposure time, and the selected frequency bandwidth modify the estimated velocity value, requiring the application of a global calibration factor. It enables physiological consistency with literature-reported values. For reference, a pilot study using bidirectional laser Doppler velocimetry reported total arterial and venous retinal flow rates of 33±9.6 and 34±6.3  μL/min, respectively.^20^ Another study found a total average retinal blood flow of 44.0±13.3  μL/min in healthy subjects, including all retinal vessels with diameters above 60  μm entering the ONH.^47^ Similarly, a Doppler Fourier-domain OCT study in 10 subjects reported a mean total retinal blood flow of 45.6±3.8  μL/min, ranging from 40.8 to 52.9  μL/min,^33^ and another study reported 44.98±9.80  μL/min (range: 30.18 to 64.58) for normal eyes.^48^ The scaling factor (α=5) brought the average total retinal arterial volumetric flow rate (39  μL/min across 14 eyes). Although this adjustment does not impact the internal consistency of the repeatability and reliability assessments, it also harmonized the derived vascular indices, such as the RI, with values obtained using Doppler ultrasound in the central retinal artery.^23^^,^^49^

Quantitative accuracy in retinal hemodynamic imaging is inherently dependent on several biological and physiological parameters. Hematocrit, the proportion of red blood cells in the blood, significantly affects the scattering properties and thus the Doppler signal strength. Inter-individual differences in hematocrit may introduce variability in flow estimates, suggesting that hematocrit-adjusted calibration protocols may be required for more personalized assessments.^50^^,^^51^ Similarly, ocular pigmentation alters light absorption and scattering within retinal tissues,^52^^,^^53^ potentially influencing Doppler sensitivity and the accuracy of vascular flow metrics. Future studies should account for these individual-level variables to further refine the quantitative fidelity of LDH-based measurements.

Furthermore, accurate and reproducible estimation of these metrics heavily depends on robust retinal vessel segmentation. Manual intervention was required in this study to ensure anatomical consistency across repeated measurements. To overcome this limitation and enable large-scale, automated analysis, a machine-learning-based vessel segmentation algorithm is being developed.^54^ This advancement is expected to improve segmentation reliability, minimize operator bias, and facilitate large cohort clinical studies.

Although the current work focuses on arterial flow, expanding the methodology to analyze retinal veins will be a critical next step. Unlike arterial vessels, veins do not exhibit strong cardiac-cycle-synchronized pulsatility, making them more difficult to segment using current temporal correlation with average arterial pulse shape approaches.

Moreover, future investigations will need to examine the impact of acquisition parameters (e.g., laser power, exposure time, and spectral bandwidth) and numerical processing parameters on the accuracy and stability of hemodynamic metrics, such as vessel neighborhood signal estimation and velocity profile analysis model.^55^ Understanding how these factors influence signal quality and quantification fidelity will be essential for standardizing LDH imaging protocols across devices and institutions.

Another significant area for development lies in transitioning from offline data acquisition to real-time implementation. Although offline LDH enables high-resolution measurement, it is computationally and logistically intensive, generating several terabytes of data per session. In contrast, real-time setups are significantly faster to operate and generate lighter datasets, making them better suited for clinical workflows and large-cohort studies. As the current real-time hardware approaches sufficient frame rates, repeating the present reliability and repeatability assessments in real-time will be essential to validate scalability and clinical applicability.

Finally, future studies will aim to incorporate rheological analyses, including more detailed characterization of flow velocity profiles, pressure-resistivity relationships, and vascular compliance. These parameters could further expand the clinical utility of LDH, offering insights into both local retinal vascular status and broader cardiovascular health.

In summary, the present study supports the potential of LDH as a promising tool for high temporal-resolution, repeatable assessment of retinal blood flow and vascular function. Although current limitations (segmentation and calibration) are being addressed, the technique offers a pathway toward non-invasive, real-time hemodynamic imaging with significant potential toward translation application in cardiovascular and ocular medicine.

## 5 Conclusion

This study demonstrates that LDH provides a robust and reliable assessment of retinal arterial hemodynamics in large vessels. The mean blood volume rate and resistivity index were the most stable biomarkers, with RI showing comparable robustness whether calculated from velocity or volumetric flow, highlighting its minimal sensitivity to calibration. In contrast, metrics such as the pulsatility index and arterial stroke volume were more variable due to their dependence on precise systolic-peak timestamp detection.

Intra-day CoVs for BVR and RI were ∼18% and 11 to 13%, respectively, and mean vessel diameter exhibited excellent precision (CoV≈2 to 3%). Inter-day reproducibility remained high (BVR≈20%, RI≈9 to 10%). Importantly, the system was robust to moderate axial and lateral positional variations, with BVR and RI CoVs typically within 10 to 15%, reducing dependence on operator alignment and patient fixation.

Overall, this first robustness study of LDH displays stable, repeatable, and alignment-robust measurements of retinal blood flow and vascular function, supporting its potential as a quantitative tool for both research and clinical evaluation of retinal and systemic vascular health.

## Data Availability

The MATLAB and C++ codes used for image acquisition and processing are openly accessible, as detailed in Sec. 2.2, via the project’s GitHub repository Digital Holography. The datasets generated and analyzed during this study can be made available upon reasonable request due to their multi-terabyte size. All hardware specifications (including the optical setup and computational architecture) are provided as open-source resources at the Digital Holography website.
